# High-order brain interactions during ketamine-induced state changes: A functional marker of response in late-life treatment-resistant depression?

**DOI:** 10.1038/s41398-026-04212-1

**Published:** 2026-07-04

**Authors:** Krisha Shah, Rubén Herzog, Alan C. Swann, Brittany O’Brien, Rahul Balakrishnan, Sanjay J. Mathew, Nicholas Murphy

**Affiliations:** 1 Texas A&M University Naresh K. Vashisht College of Medicine, Department of Psychiatry and Behavioral Sciences, Bryan, TX USA; 2 Baylor College of Medicine, Menninger Department of Psychiatry and Behavioral Sciences, Houston, TX USA; 3 Department of Psychology, University of the Balearic Islands, Palma de Mallorca, Spain; 4 Instituto de Física Interdisciplinar y Sistemas Complejos (IFISC, UIB-CSIC), Campus UIB, Palma de Mallorca, Spain; 5 Michael E. DeBakey VA Medical Center, Houston, TX USA

**Keywords:** Neuroscience, Human behaviour

## Abstract

Ketamine is a fast-acting intervention for treatment-resistant depression (TRD), yet only a subset of patients show robust clinical response, and the underlying neural mechanisms remain unclear. High-order interactions (HOI) derived from multivariate information theory provide a framework for examining nonlinear dependencies among brain regions beyond pairwise connectivity. One such metric, the O-information, captures the balance between synergistic and redundant interactions across three or more variables. In this secondary analysis of a randomized, double-blind, midazolam-controlled trial (NCT02556606), we examined EEG-derived HOI in 30 late-life veterans with TRD following a single 40-minute intravenous infusion of ketamine (0.1, 0.25, 0.5 mg/kg; n = 18) or midazolam (0.03 mg/kg; n = 12). Resting state and mismatch negativity data were analyzed at baseline, 1 h, 24 h, and 7 d post-infusion. Ketamine induced temporally dynamic alterations in redundancy-dominant O-info, with maximal effects in the alpha-band at 1 h (Cohen’s d = 2.57), attenuation at 24 h that shifted toward the theta-band, and partial resurgence in beta and gamma by Day 7. Linear mixed-effects modeling identified significant group effects across most band x metric families, with the strongest effects in alpha, beta, and gamma redundancy. Greater increases in 24-hour alpha-band redundancy were associated with greater improvement in depressive symptoms at Day 7 (β = 69.31, q = 0.05). HOI metrics also tracked acute dissociative states, with several 24-hour alpha and beta features remaining positively associated with symptom severity after correction. These findings extend prior HOI work in healthy samples to a controlled TRD cohort and suggest that ketamine induces temporally structured reorganization of higher-order brain interactions, with exploratory associations to clinical outcomes.

## Introduction

Intravenous (IV) ketamine is a rapid-acting intervention for major depressive disorder (MDD), gaining clinical momentum over the past decade. Yet, response remains variable, and recent meta-analyses reveal uncertainty regarding the clinical moderators and neurobiological mechanisms that drive its potential effects [1–3]. Advancing precision treatment guidelines for ketamine therapy will require clarifying the mechanisms underlying ketamine’s initial rapid antidepressant effects and determining how these effects can be maintained and enhanced in the absence of continued dosing.

Ketamine has been shown to reduce oscillatory alpha power (7–14 Hz) and increase gamma power (30–80 Hz), reflecting local disinhibition within neuronal clusters associated with the default mode and prefrontal networks [4]. The extent of synaptic potentiation in the gamma band has been found to inversely correlate with depressive symptom severity [5–9]. These findings suggest that ketamine shifts the brain from a low-dimensional, rigid state to a higher-dimensional, more flexible configuration: one that may be more amenable to affective and behavioral modification, such as through psychotherapy. Our previous work in treatment-resistant depression (TRD) supported this framework by showing that a single subanesthetic dose of ketamine modulates the complexity of neural signaling [8], with reduced complexity in slower signals and increased complexity in faster signals. Together, these findings suggest that ketamine’s early effects give rise to dynamic cortical changes that evolve meaningfully in the hours and days following infusion.

Only about 30–50% of TRD patients [1, 10] show robust clinical response to ketamine, underscoring the need to understand how neural changes are organized to support an optimally responsive brain state. Recent advances in multivariate information theory offer a promising approach for capturing these temporo-spatial statistical structures that underlie clinical outcomes [11].

One such approach, high-order interactions (HOI), uses generalizations of mutual information to characterize multivariate dependencies among electrode combinations, as well as the dominant quality of interaction within a network. For the former, the S-information measures the overall level of interdependencies, while for the latter, the O-information measures the dominance between synergy and redundancy. Synergy refers to relationships among variables in which the whole system conveys more information than the sum of its parts, while redundancy refers to relationships in which copies of the same information are distributed throughout the whole system. In previous work, HOI revealed increased alpha-band redundancy during rest, and increased redundancy in response to standard tones during the auditory mismatch negativity (MMN) [12]. Because redundancy reflects widespread broadcasting of shared information across the brain, this prior work suggested that ketamine might reduce top-down control, thereby increasing the influence of associative and sensory cortical systems on brain hierarchy. Notably, the effects of ketamine on resting alpha were also associated with an increased sense of derealization on the Clinician Administered Dissociative States Scale (CADSS) in healthy volunteers [12]. These findings are relevant not only to models of consciousness, where altered redundancy may reflect changes in global information integration [13, 14], but also to depression, which involves disruptions in how individuals process internal thoughts and external sensory information [15, 16].

To our knowledge, no prior study has evaluated HOI as a clinical biomarker of ketamine response in MDD. Here, we hypothesize that ketamine-induced changes in brain dynamics and depressive symptoms are associated with increases in brain redundancy. HOI provides a powerful framework for characterizing the spatiotemporal organization of functional brain networks critical to therapeutic response, positioning these metrics as promising targets for precision treatment. We present a secondary analysis of EEG data collected during the peri-infusion period of a single ketamine or active placebo (midazolam) infusion in late-life veterans with TRD. Specifically, we examined HOI among EEG channels during resting and cognitively engaged states to delineate the cortical information dynamics associated with clinical improvement following a single infusion of intravenous (IV) ketamine versus midazolam. Our study design builds upon previous research [12] to develop a dynamic framework aimed at advancing the precision treatment of MDD with ketamine, while also seeking to externally validate prior HOI findings in healthy volunteers in an independent clinical sample.

## Methods

### Design and overview

This secondary analysis used EEG data from a randomized, midazolam-controlled, double-blind, multi-arm trial investigating dose-dependent ketamine effects in TRD [ClinicalTrials.gov: NCT02556606]. Here, we aimed to characterize temporally evolving changes in higher-order brain interactions following ketamine infusion. Detailed trial methods are described in [5].

Based on prior work demonstrating dose-invariant EEG metrics [5], HOI metrics were examined using a dose-inclusive ketamine group (0.1, 0.25, 0.5 mg/kg) and compared with midazolam (0.03 mg/kg). The ketamine group included three participants at 0.1 mg/kg, five at 0.25 mg/kg, and ten at 0.5 mg/kg. Formal dose-sensitivity analyses were subsequently performed to evaluate whether pooling across ketamine doses was appropriate.

Analyses focused on EEG data collected at baseline, 1 h, 24 h, and 7 days post-infusion, corresponding to timepoints of primary clinical interest [8, 17]. HOI metrics included O-information (O-info) and S-information (S-info). O-info quantifies the balance between redundancy- and synergy- dominated interactions, whereas S-info reflects the overall magnitude of higher-order interdependencies.

Missing data were excluded from the analyses. To characterize treatment-related change, post-infusion HOI values were expressed as change scores relative to baseline (ΔHOI). These ΔHOI metrics were related to: (1) Percent change in Montgomery-Åsberg Depression Rating Scale (MADRS) scores from baseline to Day 7 (primary outcome), and (2) Absolute change in CADSS scores from baseline to 1 h post-infusion (acute dissociation).

Descriptive statistics for MADRS and CADSS were computed by arm and timepoint. This change-score framework reduced between-subject baseline variability, and the selected EEG timepoints captured acute, subacute, and sustained treatment effects.

### Participants

Demographics and clinical characteristics are presented in Table 1. Thirty-three U.S. military veterans aged ≥55 years meeting criteria for TRD were enrolled. All participants met criteria for a current major depressive episode based on the Mini-International Neuropsychiatric Interview (MINI) 7.0 [18], ≥2 failed antidepressant trials (confirmed by the Antidepressant Treatment Response Questionnaire [ATRQ] [19]), and moderate-to-severe symptom burden (MADRS [20] ≥ 27). Patients with a Mini-Mental State Examination (MMSE) [21] score <25 were excluded due to the risk of cognitive impairment. Detailed inclusion and exclusion criteria are described in Supplement Table S1.

**Table 1:** Demographics and Clinical Characteristics.

Demographics	Ketamine (n = 18)	Midazolam (n = 12)
Age in years, Mean (SD)	61.8 (5.7)	63 (5.5)
Male, n (%)	12 (66.7%)	8 (66.7%)
**Race**		
White, n (%)	9 (50%)	6 (50%)
Black, n (%)	9 (50%)	6 (50%)
**Ethnicity**		
Hispanic, n (%)	2 (11.1%)	1 (8.3%)
Non-Hispanic, n (%)	16 (88.9%)	11 (91.7%)
Weight in kg, Mean (SD)	91.6 (15.4)	89.9 (16.6)
BMI, Mean (SD)	31.1 (5.6)	29.7 (4.3)
Baseline pre-infusion MADRS, Mean (SD)	33.9 (2.9)	35 (5.1)
Day 7 MADRS, Mean (SD)	15.94 (13.2)	15.57 (21.3)
Baseline pre-infusion CADSS, Mean (SD)	0.6 (1.62)	0 (0)
1 h CADSS, Mean (SD)	15 (18.9)	4 (6.3)

*BMI*, Body Mass Index; *MADRS*, Montgomery-Åsberg Depression Rating Scale; *CADSS*, Clinician-Administered Dissociative States Scale.

This trial was approved by the Baylor College of Medicine Institutional Review Board and the Michael E. DeBakey VA Medical Center Research & Development Committee.

The final sample included 18 ketamine- and 12 midazolam-treated participants at baseline. Attrition occurred at post-infusion timepoints (ketamine: n = 16 at 24 h, n = 15 at Day 7; midazolam: n = 10 at 1 h/24 h, n = 9 at Day 7).

### Infusion protocol

**Fig. 1:**
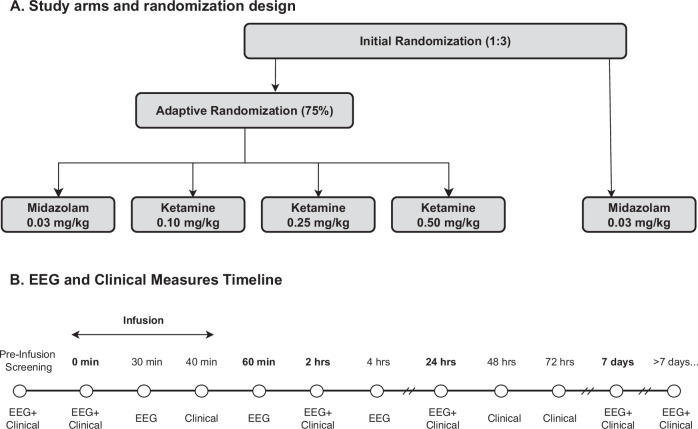
Overview of randomization design and assessment timeline. **(A)** Schematic of randomization and treatment arms. Participants were initially randomized in a 1:3 ratio to midazolam (0.03 mg/kg) or ketamine (0.10, 0.25, or 0.50 mg/kg). Subsequent participants were allocated via response-adaptive randomization (75%), favoring more effective treatment arms based on interim outcomes. **(B)** Timeline of drug infusion, EEG recordings, and clinical assessments. EEG/clinical measures were acquired pre-infusion, at baseline (0 min), and at 30 min, 40 min, 60 min, 2 h, 4 h, 24 h, 48 h, 72 h, 7 days, and >7 days post-infusion. For this analysis, resting-state EEG data were examined at baseline, 1 h, 24 h, and Day 7, while mismatch negativity (MMN) responses were analyzed at baseline, 2 h, and Day 7. Timepoints were selected to capture acute and sustained post-infusion effects.

Participants completed a standardized protocol following overnight fasting. Pre-infusion baseline assessments included clinical ratings (MADRS, QIDS-SR), 64-channel resting-state EEG, and blood sampling. Participants were randomized via Bayesian adaptive allocation [5] to receive a 40-minute intravenous infusion of either ketamine (0.1, 0.25, or 0.5 mg/kg) or midazolam (0.03 mg/kg). Post-infusion, EEG recordings were obtained at 30 min (during infusion), 1 h, 2 h, 4 h, 24 h, and 7 days, while clinical scales were repeated at 40 min, 2 h, 4 h, 24 h, 48 h, 72 h, and 7 days. Vital signs were monitored every 15 min for 4 h. An overview of the randomization schema and assessment timepoints is provided in Fig. 1 and presented in detail in previous literature [5, 22].

### EEG acquisition and preprocessing

EEG was recorded using a 64-channel Curry 7 system (SynAmps2 amplifier) with an analog-to-digital conversion rate of 1000 Hz. Resting-state recordings consisted of alternating 2-minute eyes-open and eyes-closed segments (total duration: 4 min), with eyes-closed always recorded first and no counterbalancing across visits or participants.

Both conditions were retained during preprocessing to improve artifact identification during Independent Component Analysis (ICA). After artifact removal, analyses were restricted to the first 100 s of data to isolate the eyes-closed condition and minimize visual input-related variability.

Continuous data were band-pass filtered (1–50 Hz, finite impulse response) and down-sampled to 250 Hz. Line noise (59–61 Hz) was regressed out using the CleanLine plugin. We removed artifacts using a two-step approach to differentiate spontaneous mechanical artifacts from biological artifacts (electrocardiogram/electrooculogram/blinks). First, artifact subspace reconstruction (ASR [23]) was applied to identify bad channels and recover cortical activity obscured by bursts of sporadic artifacts (channel criterion r = 0.75; burst threshold: 25 SD). Spherical interpolation was used to reconstruct excluded channels, and the dataset was re-referenced to the grand average. Second, ICA was used to identify and remove components with spatial, temporal, and spectral properties indicative of ocular and cardiac activity. The FastICA algorithm [24] was applied using the simultaneous decomposition method and dimension reduction to match the dataset rank. ICLabel [25] was used to remove components with a 90% probability of being artifacts.

Resting state data were then band-pass filtered to create individual time series for theta (4–7 Hz), alpha (8–12 Hz), beta (12–30 Hz), and gamma (30–40 Hz) bands, consistent with Herzog et al [12].

MMN data were epoched from −100 to 500 ms relative to stimulus onset and preprocessed similarly, with additional probabilistic rejection of trials exceeding ± 5 SD. Stimulus parameters and trial counts followed prior work [26]. Epoched MMN data were concatenated into whole-band continuous time series for standard and deviant conditions. During preprocessing, an average of 6 s (±18 s) of unrecoverable burst activity was removed following ASR. ICLabel rejected an average of 8.9 components (±5.7). For MMN data, an average of 36.2 (±66.3) standard trials and 3.5 (±7.2) deviant trials were excluded. These preprocessing steps were implemented to balance artifact removal with retention of sufficient data for reliable HOI estimation.

### High-order interactions metrics

We applied previously established high-order information-theoretic metrics [11] to assess nonlinear, multivariate EEG interactions. The original methodology utilized 16 electrodes [12]; however, our montage did not include FCz or a suitable substitute due to the placement of the ground and reference electrodes. To maintain methodological consistency with prior work, analyses were performed on a reduced 15-electrode subset derived from a 64-channel recording. From these 15 electrodes, we computed all the possible combinations of interacting electrodes (n-plets), ranging from 2-plets (pairwise) to a single 15-plet (global interaction). Each n-plet, representing a unique combination of n electrodes, was analyzed using four entropy-based metrics [12].

The calculus of HOI is described in detail in previous work [12]. We present a paraphrased version of this description below for ease of access. Let ${X}^{n}$ = (${X}_{1}$, …, ${{X}}_{n}$) be a set of n electrodes, where ${X}_{1}$, ${X}_{2}$, and ${X}_{n}$ correspond to the time series of electrodes 1, 2, and n, respectively. The TC, DTC, O-info, and S-info are generalizations of the mutual information, and are defined as follows:

1 $${TC}({X}^{n})={\sum }_{i=1}^{n}H({X}_{i})-H({X}_{1},...,{X}_{n})$$

2 $${DTC}({X}^{n})=H({X}_{1},...,{X}_{n})-{\sum }_{i=1}^{n}H({X}_{i}|{{X}^{n}}_{-i})$$

3 $$O({X}^{n})={TC}({X}^{n})-{DTC}({X}^{n})$$

4 $$S({X}^{n})={TC}({X}^{n})+{DTC}({X}^{n})$$

where $H({X}_{1},...,{X}_{n})$ is the joint Shannon’s entropy of the n electrodes, $H({X}_{i})$ the entropy of the i-th electrode, and $H({X}_{i}|{{X}^{n}}_{-i})$ is the entropy of the i-th electrode conditioned by the activity of all the remaining electrodes, or “residual entropy”${X}_{i}$. Estimations were performed using the Gaussian copula approximation [11, 27] using the THOI package [28].

This approach enabled comprehensive analysis of both pairwise and high-order connectivity changes across critical post-infusion timepoints relative to baseline (1 h, 24 h, 7 d). To maintain statistical power, electrode and frequency-band selection were optimized using effect size-based feature selection (see below). The reduced montage ensured both methodological continuity and scalability for the longitudinal clinical dataset.

### Dose sensitivity analysis

To assess whether HOI features varied as a function of ketamine dose (0.1, 0.25, 0.5 mg/kg), we conducted ketamine-only analyses at each timepoint (1 h, 24 h, Day 7) for resting state and mismatch negativity data. For each selected feature, linear models of the form:

$${{Feature}}_{i}={\beta }_{0}+{\beta }_{1}\cdot {{Dose}}_{i}+{\epsilon }_{i}$$

were fit, with dose treated as a continuous predictor.

Given the modest sample size (n = 18), nonparametric bootstrap confidence intervals (5000 resamples) were computed for each slope, and empirical *p*-values were derived. False Discovery Rate (FDR) correction (Benjamini-Hochberg) was applied within each timepoint across all tested features. In the absence of significant dose effects, analyses were conducted on pooled ketamine data.

Because n-plets were selected in a data-driven manner to maximize observed effects within a modest sample, subsequent analyses were interpreted as exploratory.

### Effect size computation

#### Resting state (RS)

To quantify treatment-related group differences, we computed standardized effect sizes (Cohen’s d for independent groups) between the ketamine and midazolam groups at each post-infusion timepoint (1 h, 24 h, Day 7). For each HOI metric and frequency band (theta, alpha, beta, gamma), per n-plet effect sizes were calculated as:

$$d=\frac{{\bar{X}}_{{ketamine}}-{\bar{X}}_{{midazolam}}}{{{SD}}_{{pooled}}}$$

where ${\bar{X}}_{{ketamine}}$ and ${\bar{X}}_{{midazolam}}$ represent the mean HOI value of a given n-plet across participants within each group, and ${{SD}}_{{pooled}}$ reflects pooled variance across groups. Missing, zero, and infinite values were excluded.

Effect sizes were ranked within each band x metric x timepoint, and the n-plet with the maximal absolute effect size was selected. Corresponding participant-level HOI values and electrode configurations were extracted for downstream analyses.

#### Mismatch negativity (MMN)

An analogous procedure was applied to MMN-derived time series. HOI metrics were computed separately for deviant and standard continuous data at 2 h and Day 7. After exclusion of invalid values, maximal effect size n-plets were identified and extracted at the participant level. This approach isolated condition-specific effects while maintaining consistency with resting-state analyses.

### Feature selection and model-based evaluation

Selected maximal n-plets from the effect size analysis were carried forward for model-based evaluation. For each timepoint, the n-plet exhibiting the largest absolute effect size within each band x metric combination was identified.

To evaluate robustness and account for covariates, we implemented a two-stage modeling approach. First, candidate fixed-effects structures were compared using backward elimination and exhaustive model selection based on the Bayesian Information Criterion (BIC), considering age, sex, BMI, baseline HOI, group, time, and group x time interactions. The optimal fixed-effects structure was then incorporated into a linear mixed-effects model, with subject-level random intercepts to account for repeated measures.

To assess temporal stability, n-plets with the highest effect size at each timepoint were tracked across all three timepoints. We additionally characterized distributions across bands and metrics using summary statistics (mean, median, proportion of positive vs negative effects) and visualized temporal stability using violin plots.

### Associations between HOI metrics and clinical outcomes

To examine associations between HOI metrics and clinical outcomes (MADRS and CADSS), we applied a data-driven correlation framework designed to identify candidate HOI features associated with clinical change independent of the effect-size-based feature selection procedure. For each band x metric x timepoint, Spearman correlations were computed between ΔHOI and changes in clinical scores (ΔMADRS: baseline to Day 7; ΔCADSS: baseline to 1 h). Statistical significance thresholds were derived using permutation testing (10,000 iterations), generating null distributions via random shuffling of clinical outcomes. Two-tailed significant thresholds were defined at the 2.5th and 97.5th percentiles of the permutation distribution, preserving both positive and negative effects. Surviving correlations were subjected to false discovery rate (FDR) correction using the Benjamini-Hochberg procedure (p < 0.05) [29]. For each timepoint, n-plets exhibiting the strongest positive and negative FDR-surviving associations were identified and extracted at the participant level.

### Model-based inference of clinical associations

To evaluate whether identified HOI features independently predicted clinical outcomes, we fit ordinary least squares (OLS) models with ΔHOI as the primary predictor and age, sex, BMI, and baseline HOI as covariates. Although ΔHOI captures within-subject changes, individual variability in baseline network architecture, such as baseline synergy or redundancy, may influence its relationship with clinical outcomes. Prior work suggests that baseline electrophysiological features, including gamma, theta, and alpha activity, may moderate treatment response to ketamine, supporting adjustment for baseline HOI [30, 31]. Sensitivity analyses excluding baseline HOI were conducted to assess potential collinearity effects.

## Results

### Ketamine administration induced temporally dynamic alterations in resting-state high-order interactions

**Fig. 2:**
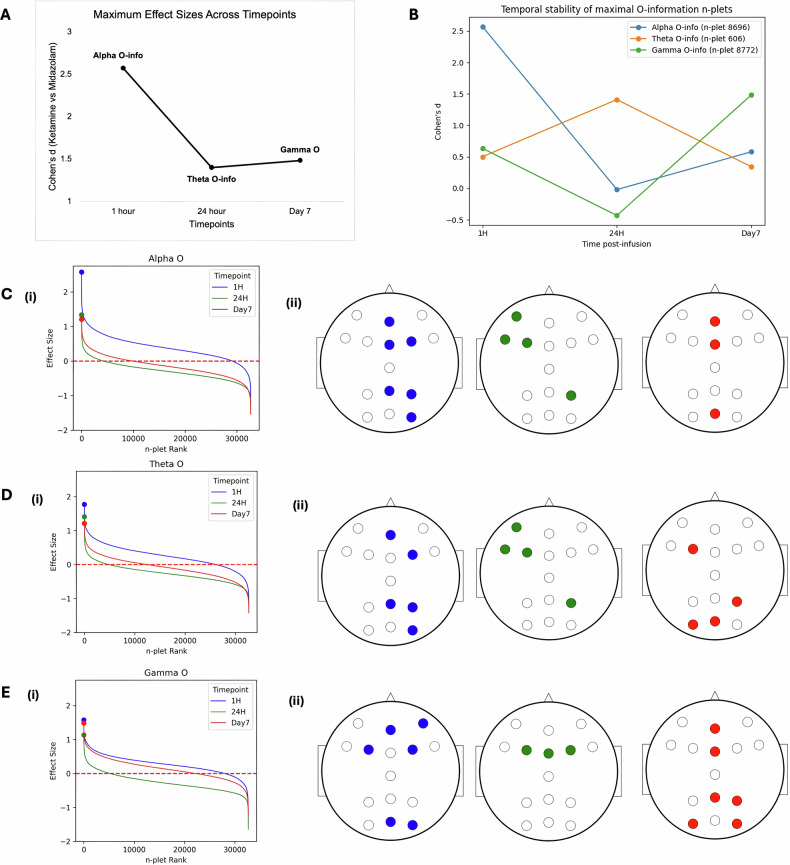
Time-dependent changes in resting-state O-information following ketamine infusion. **(A)** Maximal O-info effect sizes (Cohen’s d) peaked in the alpha band at 1 h, theta band at 24 h, and gamma band at Day 7. **(B)** Temporal stability of the maximal O-info n-plets identified at each timepoint, plotted across all three visits. **(C) - (E)** For each maximal feature: **(i)** Ranked effect sizes across all n-plets (combinations of 2–15 electrodes) at each timepoint; curves reflect 1 h (blue), 24 h (green), Day 7 (red). Filled circles: maximum effect; dashed line: zero. **(ii)** Topographical distribution of electrodes in the n-plet with the maximum effect size, plotted on the EEG 10–20 layout. Colors match the peak from panel A (blue = 1 h, green = 24 h, red = 7 d). O-info effects peaked in the alpha band at 1 h, shifted toward theta-band effects at 24 h, and showed partial gamma-band re-emergence by Day 7. Maximal n-plets showed limited temporal stability across visits. Electrode plots for all bands, metrics, and timepoints are depicted in Supplement Figure S7.

We first examined ketamine-related changes in resting-state HOI using effect-size-based feature selection followed by covariate-adjusted mixed-effects modeling. The largest single effect was observed for alpha-band O-info at 1 h (d = 2.57) (Fig. 2C(i)). Across timepoints, maximal effects followed a structured temporal progression, with alpha O-info peaking at 1 h, theta O-info at 24 h, and gamma O-info at Day 7, corresponding to peak separation at 1 h, attenuation at 24 h, and partial re-emergence by Day 7 (Fig. 2).

**Fig. 3:**
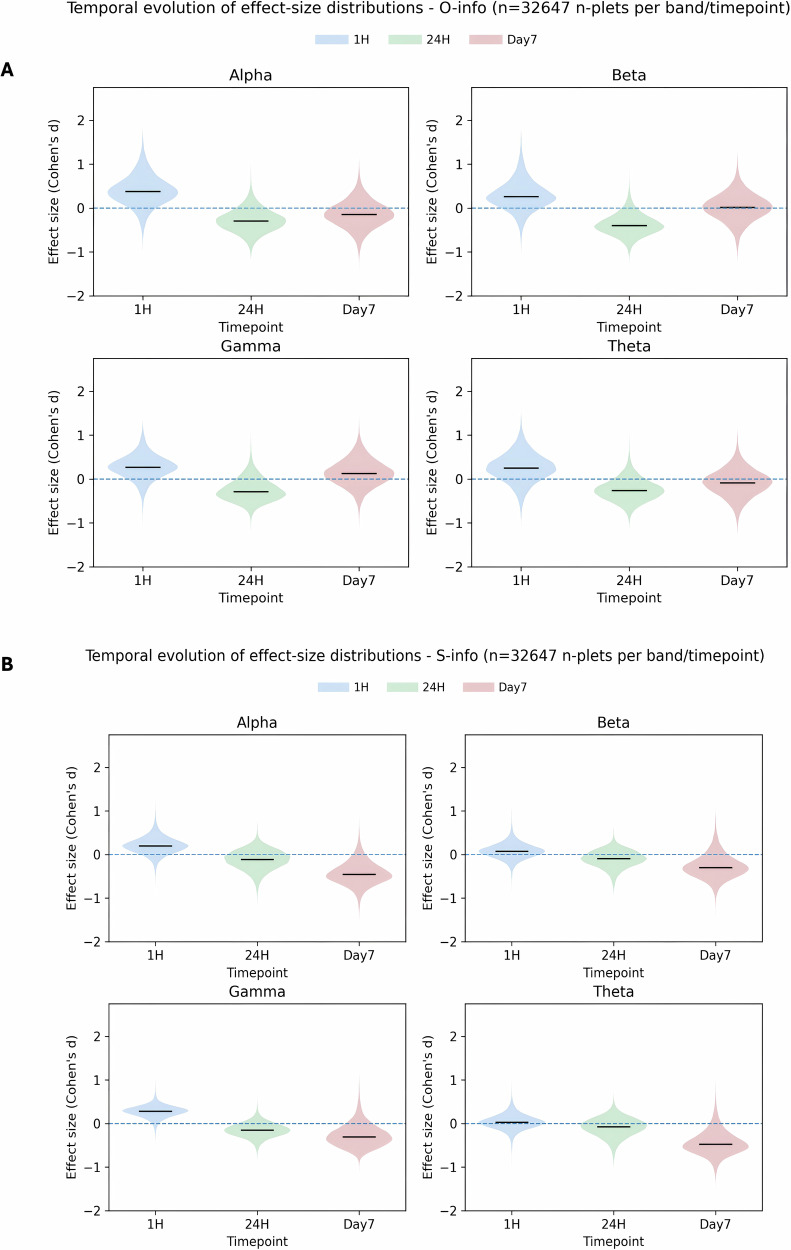
Temporal evolution of resting-state HOI effect-size distributions. **(A)** O-info **(B)** S-Info Distribution of resting-state HOI effect sizes across timepoints and frequency bands. Within each panel, violin plots show the distribution of Cohen’s *d* values across all 32,647 n-plets for alpha, beta, gamma, and theta bands at 1 h (blue), 24 h (green), and Day 7 (red). Black horizontal bars indicate medians; dashed horizontal lines indicate zero. Across bands, O-info distributions are shifted positively at 1 h, negatively at 24 h, and move toward zero by Day 7. S-info shows a similar early positive shift, with predominantly negative distributions at later timepoints, particularly in alpha and theta bands.

Distribution-level analyses across all 32,647 n-plets supported this pattern (Fig. 3). O-info showed positive median effect sizes across all bands at 1 h (median *d* = 0.25–0.38; 80–89% of n-plets positive), shifted negative at 24 h (median *d* = −0.26 to −0.40; 7–16% positive), and moved back toward zero by Day 7, with the clearest persistence in gamma (*d* = 0.13; 68% positive). S-info showed a similar early positive shift but was predominantly negative by Day 7, particularly in alpha and theta (median *d*s = −0.45 and −0.48, respectively) (Supplement Fig. S2).

Linear mixed-effects modeling showed that the best-fitting model (BIC-based) included group, timepoint, baseline HOI, and group x timepoint interaction. Significant group effects survived FDR correction in 7 of 8 band × metric families, with theta S-info not surviving correction (*q* = 0.078). Effects were strongest for alpha O-info (χ² = 48.73, *q* < 0.001), beta O-info (χ² = 24.70, *q* < 0.001), and gamma O-info (χ² = 23.41, *q* < 0.001). Baseline HOI was a significant predictor across all band × metric families after FDR correction. Significant group × timepoint effects were present for alpha O-info (χ² = 25.50, *q* < 0.001) and gamma O-info (χ² = 10.27, *q* = 0.024), indicating temporally evolving treatment effects (Supplement Table S3).

Temporal tracking of the maximal n-plets showed limited stability across timepoints, with peak configurations often attenuating or changing signs outside their defining timepoint (Fig. 2B). Spatially, peak effects shifted from midline regions at 1 h to more distributed frontoparietal and posterior configurations at later timepoints. Together, these findings suggest a dynamic reorganization of resting-state HOI after ketamine, with the clearest and most time-sensitive effects in O-info.

### Ketamine induces changes in redundancy during MMN

Second, we examined ketamine-related changes in HOI during mismatch negativity processing using effect-size-based feature selection followed by mixed-effects validation. Distribution-level analyses across all 32,647 n-plets showed a clear temporal shift (Supplement Fig. S4). At 2 h post-infusion, both O-info and S-info shifted positive across deviant and standard responses (O-info: *d* = 0.16–0.17; S-info: *d* = 0.13–0.15; 66–77% positive). By Day 7, this pattern reversed, with both metrics shifting negative (O-info: −0.28 to −0.33; S-info: −0.41 to −0.45) and a marked reduction in positive effects (2–10%) (Supplement Fig. S5).

**Fig. 4:**
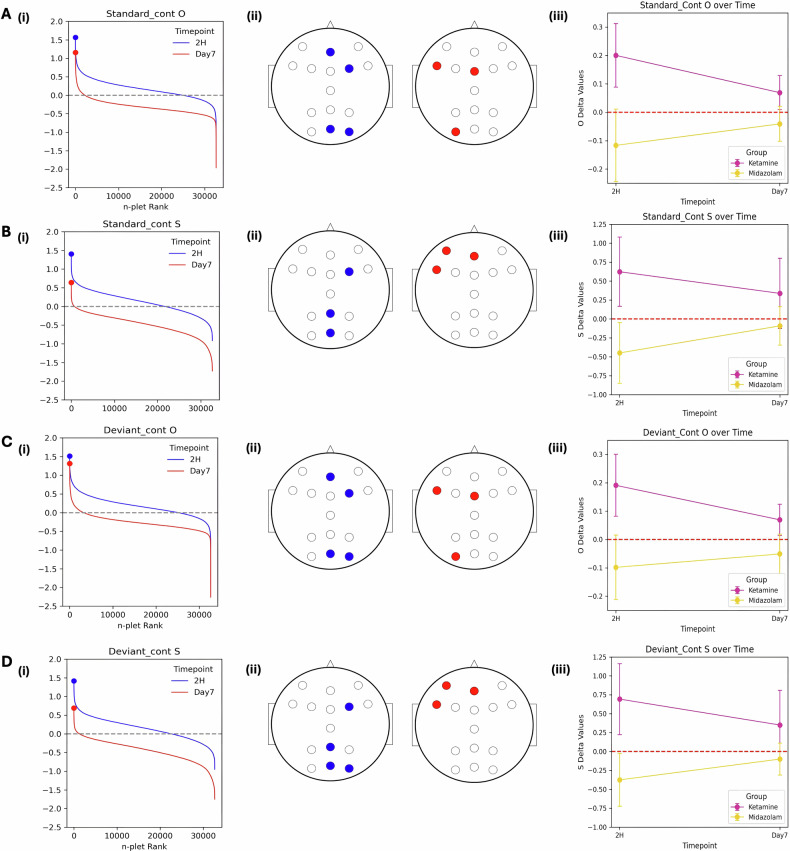
Time-dependent changes in MMN-related HOI following ketamine infusion. **(A) - (D)** For each condition and metric: **(i)** Effect sizes of each n-plet (i.e., each possible combination of 2–15 electrodes) at each timepoint; curves reflect 2 h (blue) and Day 7 (red). Filled circles: maximal effect sizes.; dashed line: zero. **(ii)** Topographical distribution of electrodes in the n-plet with the maximum effect size, plotted on the EEG 10–20 layout. Colors correspond to the peak timepoint shown in panel A (i) (blue = 2 h, red = 7 d). **(iii)** Longitudinal trajectories of participant-level O-info and S-info deltas at the maximum n-plet, stratified by treatment (pink = ketamine, yellow = midazolam; mean ± s.e.m., 95% CI). Across both standard and deviant conditions, O-info and S-info showed positive effect-size shifts at 2 h that attenuated or reversed by Day 7.

Linear mixed-effects modeling of maximal n-plet features supported a BIC-selected model including group, timepoint, and baseline HOI. Within this framework, FDR-corrected group effects remained significant for O-info in both deviant and standard responses (*q* = 0.001), whereas S-info did not survive correction (Supplement Table S6). Spatially, maximal early O-info effects were centered over central-posterior electrodes, with later configurations shifting toward more frontal-central regions (Fig. 4). Together, these findings indicate early increases followed by attenuation of task-evoked higher-order interactions, with O-info showing the most robust model-supported effects.

### HOI metrics showed selective associations with clinical outcomes following ketamine

Having established ketamine-related changes in HOI, we next tested whether ΔHOI tracked changes in clinical outcomes. Candidate features were identified using permutation-based thresholding and FDR correction, then evaluated in covariate-adjusted outcome models. Analyses were restricted to the ketamine group, as midazolam does not induce a comparable neurophysiological perturbation and associations in that condition would be expected to reflect non-specific variability rather than mechanistically meaningful coupling.

#### MADRS

**Fig. 5:**
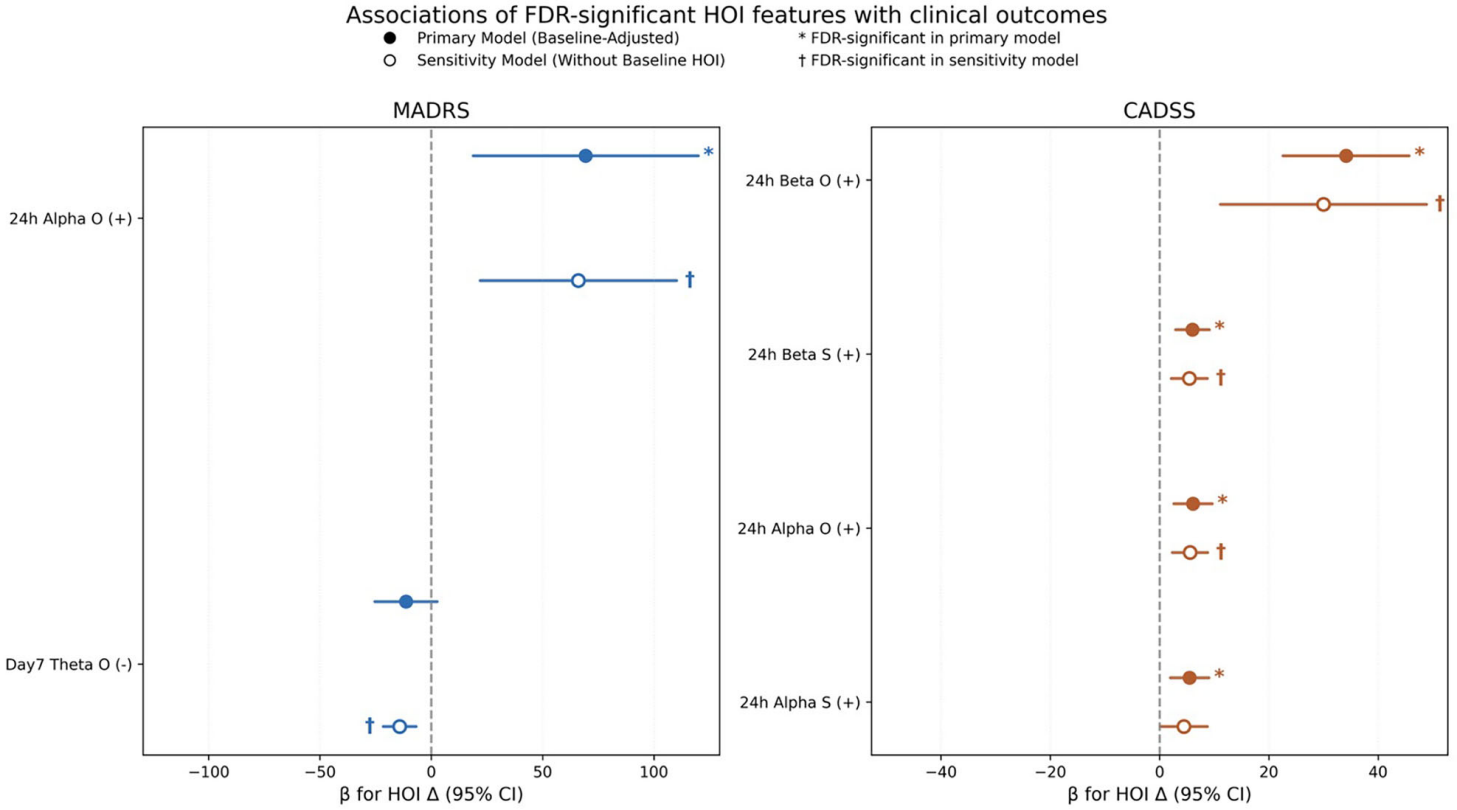
Associations of selected HOI features with clinical outcomes following ketamine. Covariate-adjusted associations between selected HOI features and clinical outcomes in the ketamine group. Left panel: MADRS Models; Right panel: CADSS models. Filled circles denote baseline-adjusted primary models; open circles denote sensitivity models excluding baseline HOI. Error bars represent 95% confidence intervals for the β estimate of ΔHOI. Asterisks (*) indicate FDR-significant effects in baseline-adjusted models, and daggers (†) indicate FDR-significant effects in sensitivity models. For MADRS, only 24-hour alpha O-info remained significant after FDR correction in the primary model. For CADSS, four 24-hour features (alpha O-info, alpha S-info, beta O-info, and beta S-info) remained significant after FDR correction in the primary model. Dissociative outcomes showed more FDR-significant HOI associations than antidepressant outcomes.

For antidepressant response, model-supported associations were limited. In the baseline-adjusted primary model, only 24 h alpha O-info remained significant after FDR correction (β = 69.31, SE = 25.77, *p* = 0.007, *q* = 0.050), indicating that greater increases in redundancy at 24 h were associated with greater Day 7 symptom improvement (Fig. 5). Several additional features showed directional effects but did not survive correction, including 24h gamma O-info (positive correlation), Day 7 theta O-info (negative correlation), and 24 h theta O-info (positive correlation) (Supplement Table S8). Thus, the MADRS findings support a selective and exploratory association, centered primarily on 24 h alpha-band redundancy, rather than a broad HOI-antidepressant signal.

#### CADSS

Associations with dissociative symptoms were stronger and more consistent. In the primary model, four 24 h features survived FDR correction: alpha O-info (β = 6.10, *q* = 0.007), alpha S-info (β = 5.48, *q* = 0.019), beta O-info (β = 34.11, *q* < 0.001), and beta S-info (β = 6.00, *q* = 0.002) (Fig. 5). All were positive, indicating that greater increases in HOI were associated with greater dissociative symptoms (Fig. 5). Earlier 1 h and 24 h features, including negative gamma-band O-info associations, were observed at the candidate-feature stage but did not survive FDR in the outcome model (Supplement Table S9).

Together, these analyses indicate that HOI-clinical associations were outcome-specific: antidepressant effects were limited and centered on a single 24 h alpha O-info feature, whereas dissociative effects showed a more robust 24 h pattern involving both O-info and S-info. Although HOI features were associated with both dissociative and antidepressant outcomes, the resulting patterns differed in timing and feature composition, suggesting that these associations are not reducible to a single shared clinical process within this dataset.

## Discussion

Our primary objective was to identify temporally evolving changes in higher-order brain interactions following ketamine and evaluate their relationship to clinical outcomes in late-life TRD. Building on previous work on HOI [12], we hypothesized that ketamine would induce temporally dynamic changes in network-level information structure, distinct from those induced by midazolam, and that these changes would relate to clinical improvement. We aimed not only to characterize the neurophysiological statistical organization associated with ketamine exposure but also to evaluate the translational potential of HOI as a clinically viable, scalable biomarker. Notably, we demonstrate these effects using a relatively low-density electrode montage, suggesting that HOI metrics may be deployable in affordable, wireless EEG systems suitable for outpatient settings.

Ketamine induced large-scale, frequency- and region- specific shifts in high-order brain dynamics. At 1-hour post-infusion, we observed widespread increases in O-info across frequency bands, peaking in the alpha range, consistent with previous results (Fig. 2A) [12]. This pattern suggests dominance of redundancy over synergy, reflecting greater shared information among distributed brain regions. Such changes may reflect reduced differentiation in neural signaling. These findings align with frameworks proposing transient reductions in hierarchical constraints under ketamine, although the present data do not directly test directional signaling. Concurrent S-info increases support for this speculation, reflecting a parallel increase in overall interdependence among electrodes as the system enters a more globally coordinated state. Distribution-level analyses across all n-plets confirmed this early positive shift, with O-info positive across all bands at 1 h and S-info showing a similar, though smaller, increase. By 24 h, both O-info and S-info shifted to negative values at the distribution level, indicating attenuation of the early redundancy-dominant state. By Day 7, effects moved back toward zero, suggesting a partial normalization effect, with the clearest persistence in gamma O-info, indicating a temporally dynamic reorganization of neural interactions after ketamine (Fig. 3).

During MMN, ketamine likewise produced early positive shifts in both O-info and S-info at 2 h across deviant and standard tones, followed by negative shifts by Day 7. However, linear mixed-effects modeling was more selective: only O-info group effects survived FDR correction, whereas S-info did not, indicating increased redundancy. Spatially, maximal early O-info effects were centered over central-posterior electrodes and later shifted toward more frontal-central sites (Fig. 4). Increased O-info suggests that circuits involved in MMN processing carried more overlapping, rather than complementary information, consistent with models proposing reduced hierarchical constraint during auditory prediction, and reinforcing previous literature [30]. By Day 7, the negative effect size shifts suggest that ketamine’s longer-term effects may selectively shift the balance between redundancy and synergy without affecting overall interaction strength (Supplementary Fig. S5**)**. Importantly, the same electrodes contributed to peak O-info for both standard and deviant conditions at the 2 h timepoint, replicating previous findings that link these scalp sites to the superior temporal gyrus, a known source of auditory processing [32].

Alpha effects warrant closer examination because they emerged as the earliest and most robust HOI changes following ketamine administration and showed the clearest association with clinical response. Prior studies have reported reductions in alpha power post-ketamine [33]. Although we did not assess power directly, our findings suggest that higher-order information structure may change independently of conventional spectral interpretations. One might expect reduced alpha power following ketamine to coincide with lower alpha redundancy- fewer oscillations and fewer overlapping signals. However, we observed the opposite: alpha redundancy increased. This dissociation between oscillatory power and redundancy is potentially important, as it suggests that HOI captures an aspect of network organization not reducible to band-limited amplitude alone. Direct comparison with spectral power will be an important next step.

The evolving effect-size trajectories (Fig. 2A), with alpha peaking at 1 h, theta at 24 h, and gamma persisting most clearly by Day 7, suggest a temporally ordered progression, with early alpha effects, intermediate theta effects, and later gamma persistence. Ketamine-associated network influence appeared to abate over time, consistent with Herzog et al. [12]. In our data, alpha-band redundancy may suggest diminished hierarchical control, potentially permitting greater local micro-circuitry flexibility (gamma band) as downstream effects increase. Importantly, however, temporal tracking of maximal n-plets showed limited stability across visits, indicating that these effects were not carried by a single fixed configuration, but rather by changing subsets of n-plets over time (Fig. 2B). Altogether, these results highlight how HOI metrics allow us to detect not just whether brain activity is connected, but how that coordination may be structured across time and frequency: a richer characterization of ketamine-related network dynamics than that provided by traditional spectral or pairwise measures.

Associations with clinical outcomes were more selective after permutation testing than those suggested by the initial candidate-feature stage. For MADRS, only 24-hour alpha O-info remained significant after FDR correction in the covariate-adjusted model, including baseline HOI, age, sex, and BMI (Fig. 5). This finding suggests that increases in alpha band redundancy at 24 h may track greater Day 7 antidepressant improvement. Other candidate associations, including 24-hour gamma O-info, were directionally informative, but did not survive correction and should therefore be interpreted as exploratory. In sensitivity models excluding baseline HOI, the same 24-hour alpha O-info remained associated with clinical improvement, while one Day 7 theta O-info feature became significant; however, the latter effect was not retained in the baseline-adjusted model and should be interpreted cautiously given marked collinearity between baseline HOI and ΔHOI.

These findings align with group-level effect size results, which showed early and prominent increases in alpha-band redundancy following ketamine. Greater channel-level redundancy, especially at 24 h, could reflect a network state more conducive to clinical recovery. Accordingly, our data support a cautious interpretation: alpha-band redundancy may be a candidate marker of antidepressant-related neural reorganization. More broadly, this pattern is compatible with frameworks such as the relaxed beliefs under psychedelics (REBUS) framework [34], which proposes that psychedelic-like states increase the importance of sensory evidence and reduce top-down influence on bottom-up signaling. In doing so, they lower the precision weighting of prior beliefs and increase sensitivity to incoming evidence, forcing internal models to update and creating a flexible neural context for therapeutic change. However, in our study, we don’t directly test that mechanism with HOI.

Associations with dissociative symptoms (CADSS change from baseline to 1 h) were stronger and more consistent. In the primary covariate-adjusted model, four 24-hour features survived FDR correction: alpha O-info, alpha S-info, beta O-info, and beta S-info, all positively associated with CADSS (Fig. 5). Thus, greater dissociation was linked to increases in network redundancy and greater higher-order interaction strength, particularly within alpha- and beta-band configurations. Notably, the strongest surviving HOI predictors of acute dissociation were observed at 24 h rather than 1 h, suggesting that post-acute network changes may retain information about earlier dissociative response. Earlier negative candidate-stage associations, including gamma-band O-info features, did not survive FDR in the outcome model.

Still, important limitations remain. Our use of a single-infusion protocol limits generalizability to repeated dosing paradigms, and the late-life, predominantly male veteran sample reduces population representativeness. Although we evaluated dose effects prior to pooling and did not observe dose-dependent relationships, the modest sample size (n = 18) limits power to detect more subtle dose heterogeneity. The relatively small midazolam arm and use of adaptive randomizations may have introduced imbalances affecting group comparisons. In addition, the reduced electrode montage, while intentionally aligned with a low-density, clinically scalable setup, limits spatial resolution and may omit combinations that contain finer-grained network structure. MMN parameters are different from prior studies [12], potentially influencing task-based HOI outcomes.

Methodologically, HOI metrics are undirected and therefore cannot resolve the directionality of information flow (e.g., top-down vs. bottom-up processes) but instead characterize how information is distributed across networks. Moreover, while we observed spatial convergence with earlier work, some topographical discrepancies suggest variability in precise electrode contributions. Finally, although our modeling framework accounted for key covariates and avoided overfitting, the combination of high-dimensional feature selection and modest sample size means that clinical associations should be interpreted as exploratory.

Future work should explicitly assess the temporal trajectories of HOI metrics under ketamine, as delineating time-dependent dynamics will be essential to understanding their clinical utility and informing dosing strategies. It will also be important to focus on refining spatial modeling of HOI dynamics, integrating HOI with complementary measures like spectral power, and assessing their predictive utility in larger, longitudinal samples.

In sum, these findings support HOI as a useful and potentially scalable framework for characterizing ketamine-related changes in higher-order brain organization. Rather than indexing only whether regions co-vary, HOI captures how information is distributed across networks over time. In this study, HOI metrics captured temporally specific changes, including early alpha-band redundancy, intermediate theta-band effects, and later gamma-band reorganization, evolving O-info effects across paradigms, and outcome-specific clinical associations, suggesting that ketamine engages large-scale circuits in a structured, non-random manner. The recurrence of frontal and central alpha redundancy across group-level and clinical analyses supports its relevance as a candidate marker of ketamine-related reorganization: sensitive to pharmacological action, temporally dynamic, and clinically relevant. Importantly, these findings extend prior work [12] by demonstrating comparable effect sizes in a midazolam-controlled depressed cohort across multiple timepoints. While further studies are needed to determine whether these reorganizations distinguish responders and remitters or predict sustained benefit, HOI-based metrics may provide a useful framework for probing ketamine’s mechanisms and informing precision treatment strategies.

## Supplementary information

Supplement Material

## Data Availability

Python codes and GitHub access are available from the research team upon reasonable request.
