# Supplementary material for: High-order brain interactions during ketamine-induced state changes: A functional marker of response in late-life treatment-resistant depression?
Source: Transl Psychiatry. 2026 Jul 4;16:374. doi: 10.1038/s41398-026-04212-1 (PMC13389161; doi:10.1038/s41398-026-04212-1)
Supplement: Supplementary file 1 — Supplement Material [file 41398_2026_4212_MOESM1_ESM.pdf]

Supplement

Supplementary Table S1: Complete inclusion and exclusion criteria

Supplementary Figure S2: Distribution-level summaries of resting-state HOI across all nplets, including median effect sizes (Cohen's d) and proportion of positive effects by band and timepoint.

Supplementary Table S3. Linear mixed-effects model results for resting-state HOI metrics

Supplementary Figure S4: Violin plot distributions of MMN HOI effect sizes (O-information and S-information) across all n-plets (n = 32,647 per condition and timepoint)

Supplementary Figure S5: Distribution-level summaries of MMN HOI across all nplets, including median effect sizes (Cohen's d) and proportion of positive effects by band and timepoint.

Supplementary Table S6. Linear mixed-effects model results for MMN HOI metrics

Supplementary Figure S7. Topographical configurations and effect-size distributions for additional resting-state HOI features across bands and metrics

Supplementary Table S8. OLS models for MADRS outcomes, including baseline-adjusted and sensitivity models. Candidate features were identified using permutation testing (10,000 iterations), thresholding, and FDR correction prior to model evaluation

Supplementary Table S9. OLS models for CADSS outcomes, including baseline-adjusted and sensitivity models. Candidate features were identified using permutation testing (10,000 iterations), thresholding, and FDR correction prior to model evaluation

Supplementary Table S1: Complete inclusion and exclusion criteria

Inclusion criteria

- Age ≥55 years
- Current MDE (Unipolar) based on the MINI 7.0
- History of ≥1 previous episode of depression prior to the current episode

(recurrent MDD) or chronic MDD (of at least two years' duration)

- Failure to respond to ≥ 2 adequate trials of FDA-approved antidepressants determined by the ATRQ criteria • QIDS-SR ≥14
- MADRS ≥ 27
- CGI-S ≥ 4
- Able to understand and sign informed consent *Exclusion criteria*
- Currently taking fluoxetine
- History of bipolar disorder, schizophrenia, schizoaffective disorder or any psychotic disorder
- Documented history of a psychotic disorder in a first-degree relative
- Current diagnosis of OCD or eating disorder
- Alcohol or substance use disorder (except nicotine) within the preceding 3 months. Clinically significant personality disorder that would, in the investigator's judgment, preclude safe study participation
- Serious and imminent suicidal or homicidal risk
- Serious, unstable medical illnesses including respiratory [obstructive sleep apnea, or history of difficulty with airway management during previous anesthetics], cardiovascular [including ischemic heart disease and uncontrolled hypertension], and neurologic [including history of severe head injury]
- Clinically significant abnormal findings of laboratory parameters [including urine ECG, toxicology screen for drugs of abuse], physical examination, or ECG
- Hypertension (systolic BP > 160 mm Hg or diastolic BP > 90 mm Hg)
- Participants with one or more seizures without a clear and resolved etiology
- Participants starting hormonal treatment in the 3 months prior to Screening
- Past intolerance or hypersensitivity to ketamine, or history of recreational use of PCP or ketamine
- Past intolerance or hypersensitivity to midazolam
- MMSE < 25 at Screening, suggesting age-related cognitive decline or mild dementia
- Ongoing use of medications with known activity at the NMDA or AMPA glutamate receptor [e.g., riluzole, amantadine, lamotrigine, memantine, topiramate, dextromethorphan, D-cycloserine], or the mu-opioid receptor
- Ongoing use of the following medications: St John's Wort, theophylline, tramadol, metrizamide
- Decrease of >25% in depressive symptoms as reflected by the QIDS-SR score from Screening to Randomization
- ECT treatment within 6 months prior to Screening
- Current VNS or rTMS therapy

MINI Mini-International Neuropsychiatric Interview.
ATRQ Antidepressant Treatment Response Questionnaire.
QIDS-SR Quick Inventory of Depressive Symptomatology-Self Report.
MADRS Montgomery Asberg Depression Rating Scale.
CGI-S Clinical Global Impression-Severity.
MMSE Mini-Mental State Examination.
NMDA N-methyl-D-aspartate.
AMPA Alpha-Amino-3-Hydroxy-5-Methyl-4-Isoxazole Propionic Acid.
ECT Electroconvulsive Therapy.
VNS Vagus nerve stimulation.
rTMS Repetitive transcranial stimulation.

Supplementary Figure S2: Distribution-level summaries of resting-state HOI across all n-plets, including median effect sizes (Cohen's d) and proportion of positive effects by band and timepoint.

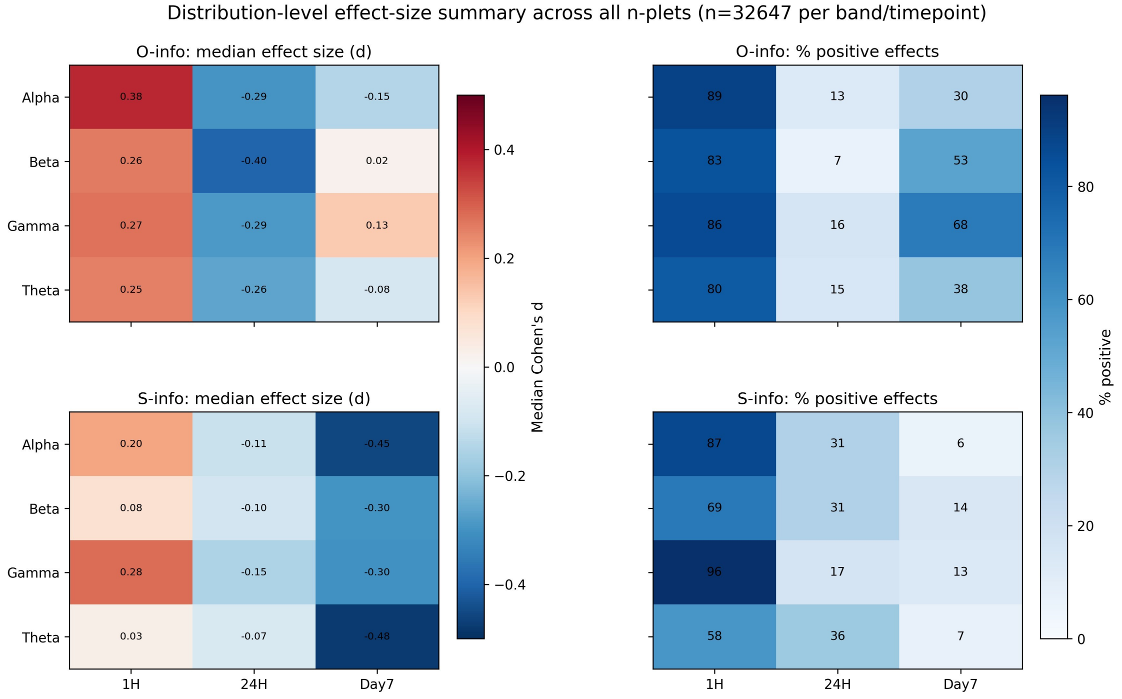

Supplementary Table S3: Linear mixed-effects model results for resting-state HOI metrics

Band	Measure	χ²_group (df)	q_group	χ²_interactio	q_interaction	q_baseline	N
Alpha	O	48.73 (3)	< 0.001	25.5 (2)	< 0.001	<0.001	30
Alpha	S	11.98 (3)	0.012	0.39 (2)	0.822	<0.001	30
Beta	O	24.7 (3)	< 0.001	4.77 (2)	0.246	<0.001	30
Beta	S	9.68 (3)	0.029	2.31 (2)	0.504	<0.001	30
Gamma	O	23.41 (3)	< 0.001	10.27 (2)	0.024	0.03	30
Gamma	S	9.12 (3)	0.032	3.92 (2)	0.281	<0.001	30
Theta	O	12.8 (3)	0.01	0.82 (2)	0.759	<0.001	30
Theta	S	6.83 (3)	0.078	1.06 (2)	0.759	<0.001	30

Supplementary Figure S4: Violin plot distributions of MMN HOI effect sizes (O-information and S-information) across all n-plets (n = 32,647 per condition and timepoint)

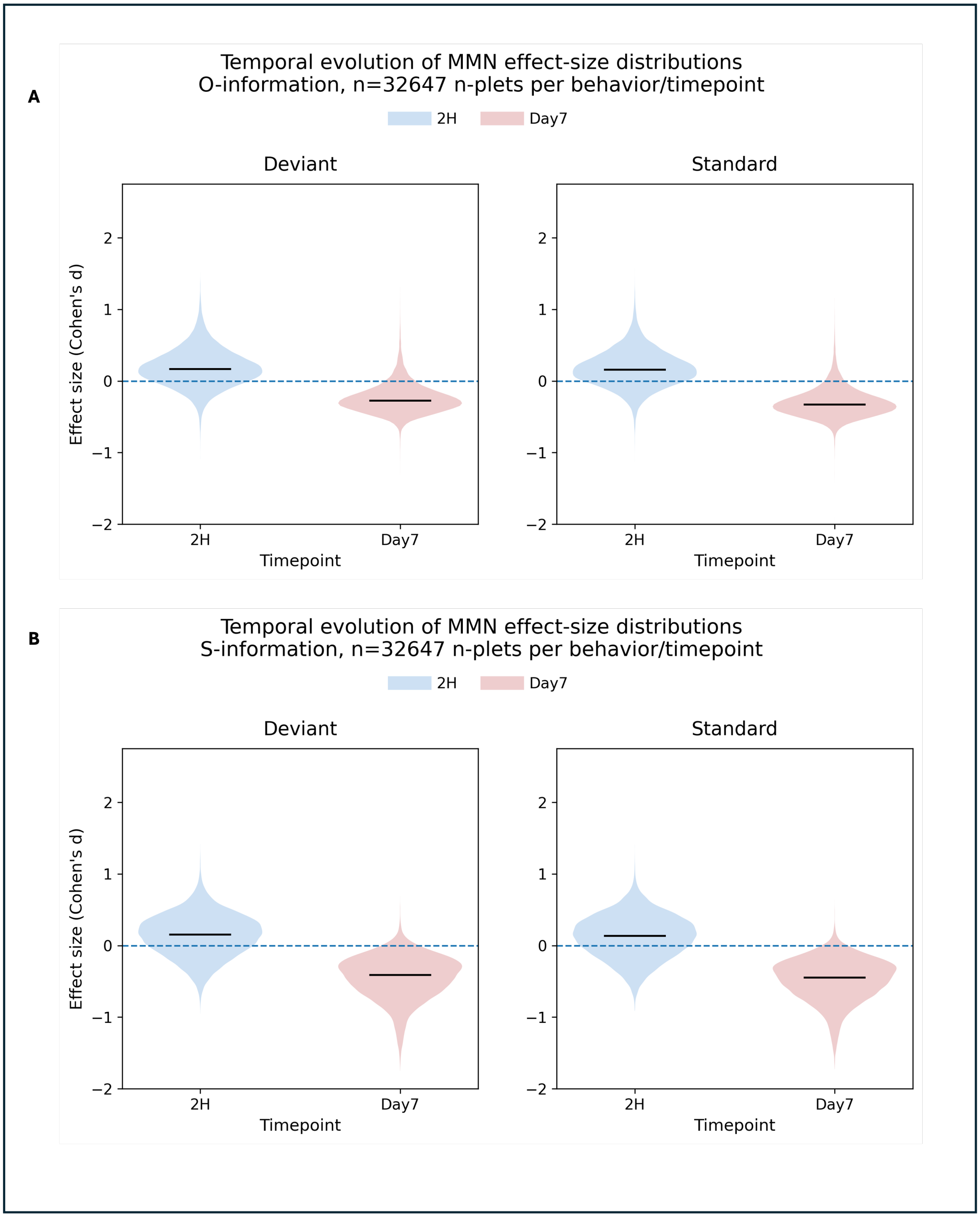

Supplementary Figure S5: Distribution-level summaries of MMN HOI across all nplets, including median effect sizes (Cohen's d) and proportion of positive effects by band and timepoint.

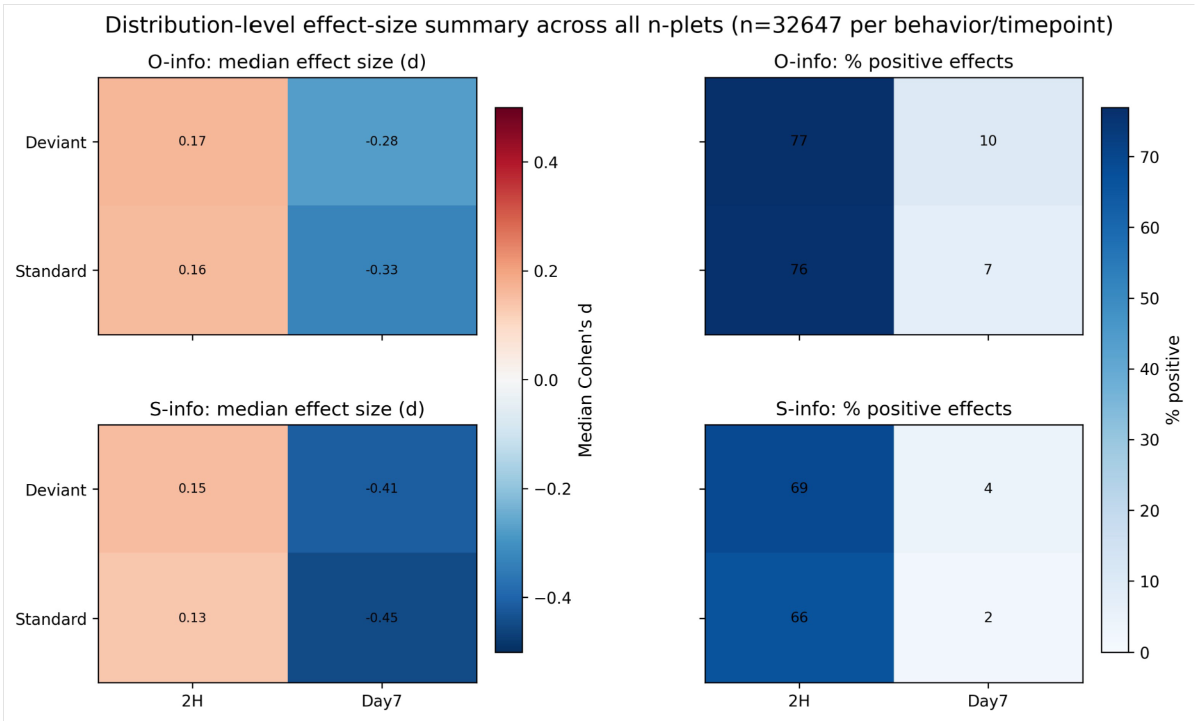

Supplementary Table S6. Linear mixed-effects model results for MMN HOI metrics

Behavior	Measure	χ^2_group (df)	q_group	q_baseline
**Deviant_cont**	O	11.78 (1)	0.001	0.9
**Deviant_cont**	S	3.96 (1)	0.06	0.09
**Standard_cont**	O	11.67 (1)	0.001	0.9
**Standard_cont**	S	2.58 (1)	0.11	0.02

Supplementary Figure S7. Topographical configurations and effect-size distributions for additional resting-state HOI features across bands and metrics

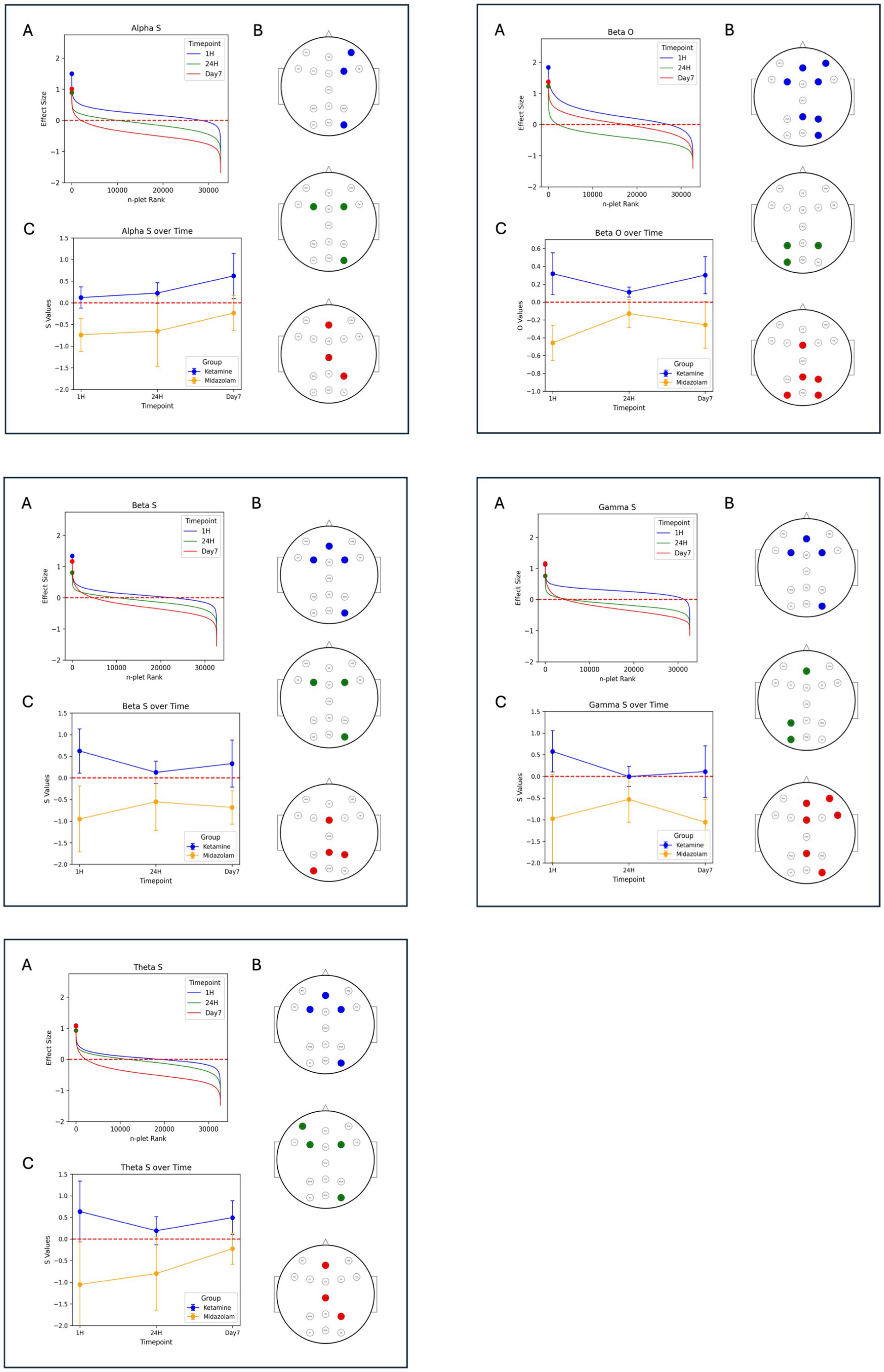

Supplementary Table S8. OLS models for MADRS outcomes, including baseline adjusted and sensitivity models. Candidate features were identified using permutation testing (10,000 iterations), thresholding, and FDR correction prior to model evaluation

Feature	N	β (Baseline Adjusted)	SE	95% CI	p	p (FDR)	Adjusted R^2 (Baseline Adjusted)	β (Without Baseline)	SE	95% CI	p	p (FDR)	Adjusted R^2 (Without Baseline)
**24h Alpha O (+)**	16	69.31	25.77	[18.80, 119.83]	0.007	0.05	0.53	66.02	22.49	[21.94, 110.09]	0.003	0.012	0.56
**24h Gamma O (+)**	16	27.07	14.54	[-1.44, 55.57]	0.063	0.219	0.24	11.66	6.26	[-0.60, 23.92]	0.062	0.145	0.12
**Day7 Theta O (-)**	15	-11.38	7.12	[-25.34, 2.58]	0.11	0.257	0.57	-14.21	3.78	[-21.62, -6.81]	< .001	0.001	0.59
**24h Theta O (+)**	16	63.04	63.44	[-61.30, 187.38]	0.32	0.561	0.21	70.1	62.03	[-51.48, 191.68]	0.258	0.362	0.23
**Day7 Gamma S (+)**	15	2.15	4.87	[-7.40, 11.70]	0.659	0.854	0.03	1.58	4.1	[-6.47, 9.62]	0.701	0.818	0.11
**24h Gamma O (-)**	16	-128.82	376.16	[-866.09, 608.45]	0.732	0.854	0.15	-315.31	189.07	[-685.88, 55.27]	0.095	0.167	0.12
**Day7 Beta O (-)**	15	127.78	1037	[-1904.74, 2160.31]	0.902	0.902	0.41	-92.19	485.12	[-1043.02, 858.64]	0.849	0.849	0.35

Supplementary Table S9. OLS models for CADSS outcomes, including baseline adjusted and sensitivity models. Candidate features were identified using permutation testing (10,000 iterations), thresholding, and FDR correction prior to model evaluation

Feature	N	β (Baseline Adjusted)	SE	95% CI	p	p (FDR)	Adjusted R^2 (Baseline Adjusted)	β (Without Baseline)	SE	95% CI	p	p (FDR)	Adjusted R^2 (Without Baseline)
1h Alpha S (-)	18	-2	2.73	[-7.35, 3.36]	0.465	0.743	0.05	-2.19	2.54	[-7.16, 2.78]	0.388	0.543	0.12
1h Beta O (-)	18	-28.5	49.09	[-124.71, 67.71]	0.561	0.743	0.06	-13.03	35.51	[-82.63, 56.57]	0.714	0.778	0.08
1h Beta O (+)	18	-2.16	33.13	[-67.10, 62.78]	0.948	0.957	-0.01	4.58	9.87	[-14.77, 23.94]	0.642	0.751	0.05
1h Beta S (-)	18	-30.77	16.56	[-63.23, 1.69]	0.063	0.304	0.19	-31.02	15.45	[-61.30, -0.73]	0.045	0.099	0.25
1h Beta S (+)	18	1.15	11.36	[-21.12, 23.42]	0.919	0.957	0.16	3.24	15.51	[-27.16, 33.64]	0.835	0.835	0.05
1h Gamma O (-)	18	-136.17	56.01	[-245.94, -26.39]	0.015	0.105	0.26	-116.97	33.27	[-182.18, -51.76]	< .001	0.004	0.28
1h Gamma O (+)	18	20.66	38.94	[-55.66, 96.98]	0.596	0.743	0	11.15	32.72	[-52.98, 75.28]	0.733	0.778	0.06
1h Theta O (-)	18	-21.09	40.86	[-101.18, 58.99]	0.606	0.743	0.19	-22.8	8.91	[-40.27, -5.33]	0.011	0.033	0.25
1h Theta O (+)	18	4.77	12.79	[-20.29, 29.83]	0.709	0.8	0.03	6.9	12.27	[-17.15, 30.94]	0.574	0.717	0.07
1h Theta S (-)	18	-6.51	6.72	[-19.68, 6.67]	0.333	0.718	0.17	-3.16	6.82	[-16.53, 10.21]	0.643	0.751	0.08
1h Theta S (+)	18	3.68	4.08	[-4.32, 11.68]	0.367	0.718	0.31	6.21	5.17	[-3.92, 16.34]	0.23	0.365	0.21
24h Alpha O (-)	16	-6.95	16.01	[-38.33, 24.44]	0.664	0.775	0.22	-10.27	12.92	[-35.61, 15.06]	0.427	0.553	0.11
24h Alpha O (+)	16	6.1	1.78	[2.60, 9.59]	< .001	0.007	0.79	5.55	1.64	[2.34, 8.77]	< .001	0.005	0.78
24h Alpha S (+)	16	5.48	1.79	[1.97, 9.00]	0.002	0.019	0.76	4.45	2.17	[0.19, 8.71]	0.041	0.099	0.72
24h Beta O (-)	16	4.85	90.78	[-173.09, 182.78]	0.957	0.957	0.12	-47.36	47.33	[-140.12, 45.41]	0.317	0.462	0.14
24h Beta O (+)	16	34.11	5.88	[22.58, 45.65]	< .001	< .001	0.88	29.97	9.62	[11.12, 48.83]	0.002	0.008	0.86
24h Beta S (+)	16	6	1.57	[2.92, 9.07]	< .001	0.002	0.82	5.42	1.68	[2.12, 8.72]	0.001	0.007	0.8

**24h Gamma O (-)**	16	-297.77	218.94	[-726.90, 131.35]	0.174	0.553	0.28	-103.97	129.07	[-356.94, 149.00]	0.42	0.553	0.1
**24h Gamma O (+)**	16	4.77	3.43	[-1.96, 11.49]	0.165	0.553	0.42	4.88	3.05	[-1.10, 10.86]	0.11	0.202	0.47
**24h Gamma S (+)**	16	5.45	4.94	[-4.23, 15.14]	0.27	0.684	0.29	5.11	2.56	[0.11, 10.12]	0.045	0.099	0.35
**24h Theta O (-)**	16	-55.46	28.27	[-110.87, -0.06]	0.05	0.29	0.46	-28.97	12.7	[-53.85, -4.08]	0.023	0.061	0.46
**24h Theta O (+)**	16	6.88	5.34	[-3.58, 17.34]	0.198	0.576	0.51	5.32	3.14	[-0.84, 11.49]	0.091	0.176	0.51
**24h Theta S (+)**	16	5.04	2.77	[-0.40, 10.48]	0.069	0.304	0.58	3.69	3.44	[-3.05, 10.43]	0.283	0.431	0.57
**Day7 Alpha O (-)**	15	-90.43	92.25	[-271.24, 90.38]	0.327	0.718	0.48	-31.61	10.58	[-52.34, -10.88]	0.003	0.01	0.39
**Day7 Alpha O (+)**	15	49.58	31.37	[-11.89, 111.06]	0.114	0.443	0.59	49.06	16.08	[17.54, 80.58]	0.002	0.009	0.63
**Day7 Alpha S (+)**	15	2.37	2.64	[-2.80, 7.54]	0.369	0.718	0.45	3.07	0.56	[1.98, 4.17]	< .001	< .001	0.49
**Day7 Beta O (-)**	15	-42.25	59.28	[-158.44, 73.95]	0.476	0.743	0.35	-44.89	29.54	[-102.78, 13.00]	0.129	0.214	0.41
**Day7 Beta O (+)**	15	30.29	48.2	[-64.18, 124.77]	0.53	0.743	0.34	43.49	10.59	[22.74, 64.24]	< .001	< .001	0.4
**Day7 Beta S (+)**	15	1.58	3.07	[-4.44, 7.60]	0.607	0.743	0.3	2.52	1.1	[0.36, 4.68]	0.022	0.061	0.28
**Day7 Gamma O (-)**	15	-18.07	35.99	[-88.61, 52.47]	0.616	0.743	0.02	-11.27	30.29	[-70.65, 48.10]	0.71	0.778	0.07
**Day7 Gamma O (+)**	15	1.45	12.63	[-23.31, 26.22]	0.908	0.957	0.08	3.09	14.45	[-25.24, 31.42]	0.831	0.835	0.03
**Day7 Gamma S (+)**	15	3.51	5.22	[-6.73, 13.75]	0.502	0.743	0.27	5.24	2.87	[-0.38, 10.86]	0.067	0.139	0.24
**Day7 Theta O (-)**	15	-38.88	70.18	[-176.42, 98.67]	0.58	0.743	0.31	-47.32	12.14	[-71.12, -23.53]	< .001	0.001	0.38
**Day7 Theta O (+)**	15	47.61	43.5	[-37.64, 132.87]	0.274	0.684	0.54	48.96	15.28	[19.02, 78.90]	0.001	0.007	0.59
**Day7 Theta S (+)**	15	2.49	3.97	[-5.29, 10.26]	0.531	0.743	0.54	4.45	2.85	[-1.13, 10.03]	0.118	0.207	0.5
